# A targeted interprofessional educational intervention to address therapeutic adherence of venous leg ulcer persons (TIEIVLU): study protocol for a randomized controlled trial

**DOI:** 10.1186/s13063-019-3333-4

**Published:** 2019-04-29

**Authors:** Sebastian Probst, Lara Allet, Jocelyne Depeyre, Sophie Colin, Monika Buehrer Skinner

**Affiliations:** 1 HES-SO University of Applied Sciences and Arts Western Switzerland, School of Health Sciences, Geneva, Switzerland; 2 University Hospital, Geneva, Switzerland; 3 University of Zurich, Epidemiology, Biostatistics and Prevention Institute, Zurich, Switzerland

## Abstract

**Background:**

Venous leg ulcers are slow-healing wounds with a high recurrence rate of 70% and a 60% risk of becoming chronic. Signs and symptoms such as pain or exudate are not only a burden on those affected but also on the healthcare system and society in general. The estimated leg ulcer prevalence in the general population is 1%. Treatment costs for leg ulcers are estimated to be 3% of overall health expenditure. Current therapeutic approaches are multifaceted and include compression therapy, leg elevation, specific ankle-exercises and a protein diet. They require an interdisciplinary team of health care professionals. Approximately 70% of patients have a knowledge deficit with regards to therapeutic measures and have difficulties with adherence to treatment protocols. Therefore, it is of utmost importance that the treatment team provides effective patient education and support during the learning phase. However, there is little evidence and no published studies that describe and evaluate effective interdisciplinary educational interventions that target compliance/adherence to the treatment plan in patients with leg ulcers. We therefore propose to develop an evidence-based interprofessional educational intervention and evaluate its feasibility first in a pilot study and subsequently in a randomized controlled trial.

**Method/Design:**

First, the development of an evidence-based educational intervention in collaboration with an expert panel is proposed and second, a randomized controlled feasibility study in a wound-care outpatient clinic. Eligible patients (*n* = 20) with leg ulcers will be randomized to receive either interdisciplinary education and usual care or only usual care, for 12 weeks. Data will be analyzed using SPSS version 25. Univariate and bivariate analysis will be conducted according to the data level and distribution of the data.

**Discussion:**

We will first develop an evidenced-based educational intervention and second, we will examine the feasibility of implementing this educational intervention in a realistic care context in patients with leg ulcers. The results will inform the final design of a subsequent randomized controlled trial, which will examine the effectiveness of the educational intervention. An intervention that enhances patient adherence to therapy would be beneficial to individual patients and to society as a whole.

**Trial registration:**

ClinicalTrials.gov, NCT03454698. Registered on 6 March 2018.

**Electronic supplementary material:**

The online version of this article (10.1186/s13063-019-3333-4) contains supplementary material, which is available to authorized users.

## Background

Venous leg ulcers (VLU) are poorly healing wounds caused by chronic venous insufficiency (CVI) [1]. CVI is a form of peripheral vascular disease that originates from an obstruction of the venous system or a defective venous valve, leading to edema, venous hypertension, skin inflammation and finally to venous leg ulcers [2]. Of all poorly healing wounds 60% are attributable to VLU [3]. Healing times are long with 60% of these wounds healing within 6 months and another 33% within 1 year. The wound never closes in 7% of cases [4]. Up to 70% of patients with leg ulcers suffer a recurrence within 3 months after wound closure [5–7]. Hence, VLU is a health problem that affects most patients for life [8].

Epidemiological data from industrialized countries including the USA and Scandinavia have shown that the prevalence of leg ulcers is 1% in the general population and 3% in people over 80 years of age [9]. An extrapolation of these findings to the Swiss context would suggests that there are an estimated 84,000 to 94,000 people suffering from VLUs, of whom 12,500 are above 80 years of age [10].

Consequently, these chronic wounds are a burden not only on the affected persons themselves, requiring expensive treatment, but also on society as a whole, as an estimated 2–3% of all health expenditure covers the treatment of this condition [9]. With a Swiss health budget of nearly 78 billion Swiss francs in 2017, this would translate into an annual cost up to 3 billion Swiss francs [11].

## Current treatment of venous leg ulcers

Standard care for the treatment and prevention of VLU includes compression therapy, elevation of the leg, exercises in the foot region, ambulating and a balanced diet rich in proteins and vitamins [12–16]. The therapeutic approach to the management of VLU is a multifaceted approach targeting the management of risk factors associated with prolonged healing times and recurrence. As patient adherence to the care plan is an essential factor in leg ulcer healing and in the prevention of recurrent ulcers, the individual components and factors associated with non-adherence are of interest. Most of the evidence on adherence originates from small studies and there are no published results from large-scale studies. The following sections reflect published evidence on measures taken to prevent recurences.

### Adherence to and patient knowledge of compression therapy

#### Adherence to compression therapy

A systematic review found that patients’ adherence to compression therapy varies widely between 10% and 80% [17]. Findings showed that non-adherence to compression therapy is associated with delayed wound healing and equates to a 2–20-fold increase in recurrence [18]. The literature cites a lack of patient knowledge of adherence to treatment as a reason for non-adherence [19–21]. For example, Edwards and colleagues [19] showed that because of these knowledge deficits, only 27% of patients with leg ulcers wear compression stockings. One reason given for patients’ knowledge deficit is lack of education by professionals during delivery the of care, due to a lack of up-to-date information and appropriate time resources to provide the required education [22].

#### Interventions to improve adherence and knowledge

Intervention studies seeking to influence adherence to compression therapy and other aspects of the treatment were carried out by Heinen and Brooks. Based on their identification of factors for non-adherence, Heinen and co-workers developed and evaluated a counselling program aimed at improving the adherence to compression therapy and physical activity in patients with leg ulcers (“Lively Legs”) [23]. Treatment adherence was surveyed using patient interviews. One of the main findings was that although an increase in adherence was observed after 18 months in both groups (from 37% to 46% in the intervention group and from 27% to 45% in the control group), no significant or relevant difference was found between the groups. These findings might first be explained by the fact that the patients’ wound status was different at baseline—overall 68% had an open wound, while in 32% of patients the wound had already closed—and second by the fact that the overall dropout rate was 26%.

An educational intervention was examined in a study by Brooks and co-workers [24]. The subjects in the control group wore the compression stockings for an average of 3.3 h per 24 h longer when compared to the intervention group. No published studies describe any successful and/or feasible interventions that increase adherence to compression treatment.

### Adherence to and knowledge of elevation of the legs and physical activity (walking and foot exercises)

#### Adherence to leg elevation and physical activity

In addition to adherence to compression therapy, the recurrence rate of leg ulcers is associated with elevating the legs to heart level for 30 min three to four times a day [25] and with regular isometric exercises. Brooks and co-workers [24] found that patients who had been educated to elevate their legs did this for 2 h longer per day than the control group, which had received no such guidance (*f* = 2.88, *p* = 0.015). A further measure described in the literature as reducing recurrence is to instruct patients to ambulate for 150 min a week (30 min, 5 days per week) at slow to medium pace to increase blood flow [26].

Isometric exercises in the ankle area are another way to prevent recurrence of leg ulcers. Van Hecke and co-workers [27] observed an increase in adherence to foot exercise regimes from 0 at baseline to 17 after 3 months (*p* = 0.003; Z = − 2.97). Kelechi and co-workers [28] observed an improvement in eversion of the right ankle by a mean of 1.3 (SD = 13.0; *p* = 0.543) following three home visits and three Skype consultations, in a small study of 21 participants.

### Adherence to and knowledge of nutritional recommendations

#### Adherence to nutritional recommendations and relationship to wound healing

The patient’s diet and nutritional status are strongly associated with wound healing. Older adults’ adherence to nutritional recommendations is often insufficient due to their comorbidities, multiple medication usage, chewing and swallowing difficulties, physical limitations, limited access to healthy foods or a lack of social support [29, 30]. Malnutrition amongst older adults in general has been described but is still unaddressed [31].

Patients with chronic wounds require higher than recommended dietary allowances of nutrients to promote wound healing due to the increased metabolism activated by chronic inflammation and increased cellular activity in the wound. Possibly there is a discrepancy between nutritional needs and food consumption, especially when it comes to protein intake [31]. Protein deficiencies slow the healing process and the formation of scars, as the formation of connective tissue and collagen synthesis requires the presence of amino acids such as arginine or cysteine [32, 33]. Patients with protein deficiency are advised to increase their daily protein intake to 1.2–1.5 g/kg body weight.

#### Education for leg ulcer patients

The literature indicates that patients with VLUs do not have sufficient knowledge of the pathophysiology of their condition or of preventive measures to perform effective self-management [1, 2, 20, 34]. Since knowledge of preventive and therapeutic measures is a prerequisite for their implementation, education and support from healthcare specialists is essential.

Successful educational interventions are described in populations with other conditions like diabetes mellitus or asthma [35, 36]. However, there are few data on the effectiveness of educational measures in patients with venous leg ulcers. In particular there are no data on educational measures targeting improvement of patients’ knowledge of the etiology/pathophysiology of their disease and their adherence to customized treatment measures. Because patients are often left on their own after their ulcer has healed and have to take sole responsibility for their leg care, this education should take place before the completion of treatment. Due to their close therapeutic relationship, wound-care specialists are predestined to offer this educational training. The education aims to improve the adherence to treatment recommendations.

The few studies available show that the recurrence rate can be reduced by up to nine times by providing education [24, 37]. O’Brien and co-workers [38] performed a qualitative study, interviewing 10 patients about their attitudes to physical exercise. The results showed that patients learnt to understand the relationship between the wound and their adherence to treatment. A randomized controlled trial showed that by educating the patient, the recurrence rate was reduced from 36% to 4% within 1 year (log rank test = 8.28, *p* = 0.004) [24]. A recent randomized controlled feasibility trial using a 12-week supervised exercise program as adjunct therapy to compression in patients with VLU showed that due to exercises the healing rate was higher in the intervention group (83% vs. 60%) [39]. There is no published evidence on the effectiveness of interprofessional interventions in patients with VLU.

### Study objectives

The aim of the protocol is (1) to develop an interdisciplinary educational intervention (nursing, physical therapy and nutrition) and (2) to evaluate the feasibility of implementing it for patients with VLUs and compare their adherence to therapy to that among patients receiving standard care. The results from this pilot study will inform the design of the main study, which will measure the effectiveness of this interdisciplinary intervention.

## Methods: participants and interventions

The TIEIVLU study is a project with two parts. First is the development of an evidence-based interprofessional patient education intervention and second is the implementation of the intervention and testing of its feasibility and acceptability.

A small-scale single-center, randomized, controlled trial is proposed as a pilot to the main study. Randomization will occur at the subject level, Feasibility and acceptability will be measured using the participation rate and the completeness of the data.

The educational intervention will be developed at the School of Health Sciences, HES-SO University of Applied Sciences and Arts, Geneva. The content of this educational tool is to improve patient’s knowledge and self-efficacy and subsequently adherence to comprehensive treatments such as compression therapy, leg elevation, physical activity and nutrition.

### Setting

The pilot study will be conducted in one outpatient wound-care clinic in western Switzerland. This outpatient wound-care center specializes in the care of patients with leg ulcers. Their staff are interested and experienced in the conduct of scientific studies. The participating outpatient wound-care center treats 45–66 patients with leg ulcers every year.

### Eligibility criteria

The following inclusion criteria apply to participants in this study:

- An existing open venous leg ulcer
- An ankle brachial pressure index (ABPI) between 0.8 and 1.3
- Age over 18 years
- Proficiency in the French language

In addition, the following exclusion criterion applies:

- Valid informed consent is not or cannot be given

### Groups

**Fig. 1:**
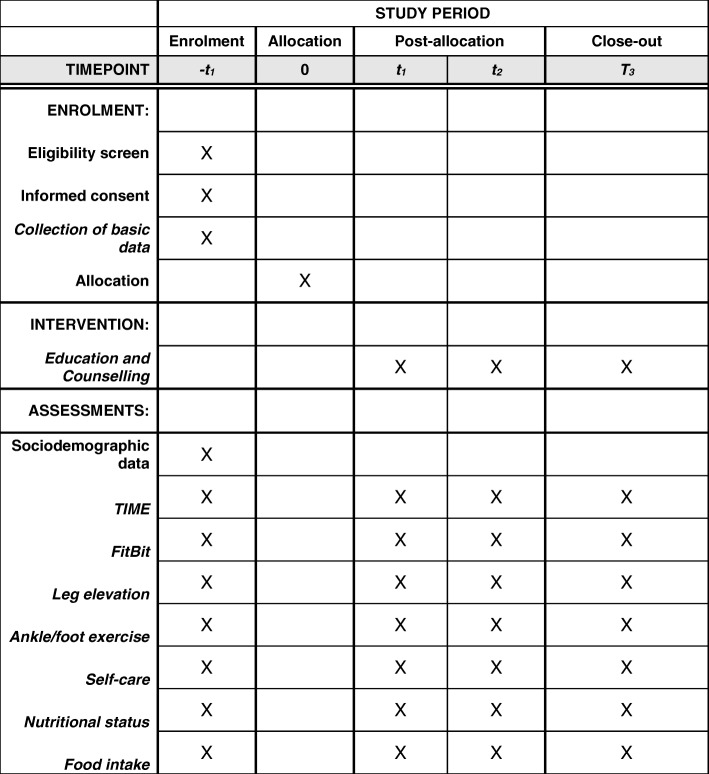
Randomization and data collection

A randomized controlled pilot trial with three measurement points will be carried out over 3 months to evaluate whether the intervention sustains an effect on the participants’ adherence to treatment. (Fig. 1). The control group (CG) will receive usual care. “Usual care” for patients in the CG is defined as follows: visits to the outpatient wound-care center as directed by a physician and wound care performed by the wound expert according to the hospital’s own standards. This standard corresponds to the one from the European Wound Management Association (EWMA).

The intervention group (IG) will receive usual care plus the educational intervention: “usual care” as described for the CG also applies to the IG. The intervention will consist of giving patients an opportunity to learn about the use of compression bandages, wearing and putting on compression stockings, physical activity, good skin care and a high-protein, vitamin-rich diet. This group will be supervised by another study nurse (not the nurse who is in charge of the control group).

### Intervention: interprofessional one-on-one educational intervention

The nurse performing the intervention will be a wound expert well-versed in the use of compression bandages, wearing and putting on compression stockings, physical activity, good skin care and a high-protein diet. The intervention nurse will conduct the educational sessions. The content of the educational sessions will be standardized and identical and will cover:

- Pathophysiology of leg ulcers
- Wearing of compression stockings
- Isometrics exercises in the region of the lower extremities
- Physical activity
- High-protein diet

The educational sessions will follow a standardized protocol.

Patients in the CG will receive usual care at the outpatient clinics. Usual care includes the T = tissue removal, I = infection control, M = moisture balance and E = edge advancement (TIME) strategy proposed by the EWMA [40]. This treatment strategy optimizes the wound-healing process by reducing edema, exudate, and bacteria in the wound.

Patients in the intervention (IG) group will be receiving the same usual care as the patients in the CG. In addition, they will receive training in the following activities at 3-month intervals over 1 year.

#### Putting on and wearing compression stockings

The pathophysiological reasons why clients should be wearing compression stockings will be explained. In addition, each patient will receive an individual demonstration of how to best put on and take off the compression stockings.

#### Exercises in the region of the lower extremities

Patients will be instructed to do regular foot and ankle exercises that can help strengthen the leg muscles and improve blood flow in the legs. While they are sitting (or standing), they have to flex their ankles by pointing their toes away from them and then pointing them up. Patients are instructed to do 10 repetitions of the exercise several times each day [41–43]. These exercises are consistent with the exercises in the guidelines for exercise and physical activity for older adults [44].

#### Physical activity

Patients will receive a wearable Fitbit. A Fitbit is an activity tracker, wireless-enabled wearable device that measures the number of steps walked. Through this technology the numbers of patients’ steps will be calculated and the patient will be reminded to move [45].

#### Elevation

Patients will be advised to elevate their feet to heart level at least 2 h per day.

#### Nutrition

Patients will be educated to eat a protein-rich diet. As with the movement, patients will also be instructed to evaluate their protein intake with the help of a nutrition log. To this end, patients will be educated on the protein content of various common foods.

### Sample

The study participants recruited will be consecutive patients of the participating institution that meet the inclusion and exclusion criteria. For this pilot study 20 patients will be recruited.

### Recruitment

Study participants will be recruited by nursing staff in the participating outpatient wound-care center. Patient lists will be screened daily for potential study participants diagnosed with venous leg ulcers. The nurses will assess the inclusion and exclusion factors for these potential participants and, if inclusion is possible, invite them to participate. Participants will be given 24 h to decide whether or not to participate. After obtaining informed consent, the participants will be referred to the study nurse, who will collect the data at baseline and at further data acquisition times along the timeline (T_0_ to T_3_). In the event that a person declines to participate, a mini-dataset will be collected comprising data on age, gender and reason for refusal. The mini-dataset will not contain health-related data. However, patients will be orally informed that stating the reason for declining to participate is entirely voluntary and that they can have the reason struck from the record at any time.

## Methods: assignment of intervention

### Randomization

After collection of baseline data, participants will be randomly assigned following simple randomization procedures (computerized random numbers) to one of two treatment groups. Gray sealed opaque numbered envelopes will be used. The intervention nurse will open the envelope and allocate the participant to the respective group.

### Blinding

Due to the intervention being an educational program, neither the participants nor the staff performing the intervention can be blinded to treatment allocation. However, the person entering and analyzing the data will be blinded to the participants’ group assignments.

## Methods: data collection, management and analysis

### Data collection

The feasibility (including acceptability) will be measured using the participation rate and the completeness and the acceptability of the intervention. The participation rate is the number of persons participating in the study divided by the number of persons eligible for participation. The completeness is measured by calculating the amount of missing data. The acceptability of the intervention is assessed from the data on self-care in patients with venous leg ulcers, physical activity and quality of life, food intake and nutritional status. The data from the intervention and control groups will be collected by each group’s own trained study nurse. Baseline data will be collected at the outpatient clinic whereas at T_1_, T_2_ and T_3_ the data will be collected during home visits.

At baseline (T_0_), basic demographic data such as age, sex, height, weight, marital status, socioeconomic factors and medical history will be recorded. Diagnostic information on the first occurrence of leg ulcers, venous interventions, deep-vein thrombosis, cardiac situation (chronic venous insufficiency (CVI) severity, Ankle Brachial Pressure Index (ABPI; ABPI = P_leg_/P_arm_), blood pressure, heart rate, edema) and other comorbidities will be obtained from the patient documentation on a single occasion. The data on self-care in patients with venous leg ulcers, physical activity and quality of life, food intake and nutritional status are obtained using instruments specifically validated for these concepts.

Health-related behavior is acquired monthly as a self-report (questionnaire) by the participants. The questionnaire enquires into the following parameters during the previous 24 h:

- Duration of wearing the compression stockings (in hours)
- Frequency and duration (time) of the elevation of the leg above heart level (minutes per day)
- Amount of physical activity (through a Fitbit)
- Number of foot exercises performed (how many days a week the foot exercises are performed)
- Nutrition (food intake assessment and nutritional status)
- Self-care

In addition, the following wound-related data are collected in patients with wounds:

- Date of recurrence
- Wound characteristics (size, time to possible healing, wound classification according to Widmer [46])
- Wound dressings used (including for compression)

### Data management

The study nurse (SN) will collect the data on the intervention and control the accuracy of the documentation. Data entry will be performed using electronic support (EvaSys software) or paper format. The data quality assessment will be conducted in a cross-over procedure among the SNs.

Data entry will be checked for logical inconsistencies, extreme outliers, missing data and distributional properties. A random sample of 10% of all data entry forms will be checked. If the error rate is higher than 5% a second data entry will be performed.

All data will be kept in a cabinet in the project manager’s office at the School of Health Sciences, Geneva. Confidentiality will be assured. Only the investigators and the designated research team will have access to the data. Patient data will be coded, and the patient’s name and unique identifiers will not appear on any data forms. Links between ID and encoding will be accessible only with password restricted to the principal investigator. The Statistical Package for Social Sciences IBM SPSS 25.0 will be used for data analysis.

### Statistical methods

#### Univariate analysis

The sample (intervention and control groups) will be described, according to the data level, in terms of their demographic and health data, using descriptive statistics. Absolute and relative frequencies will be determined for categorical variables. Numerical averages and standard deviations will be calculated for normally distributed variables. The numerical median and IQR or range will be calculated for non-normally distributed variables. Exact 95% confidence intervals will be calculated where relevant. The calculations will be performed for each data acquisition time point.

#### Bivariate analysis

To compare the groups (intervention and control) at T_0_, a parametric test (*t* test) or a non-parametric test (Mann-Whitney U test) will be used for sociodemographic and health-related data depending on the data level and distribution. All tests will be two-sided.

### Ethics approval

The study will not be started until it has been examined and improved by the Ethics Committee in the Canton of Geneva. The ethical foundations of this study are based on the “Research on Humans” guideline of the Swiss Academy of Medical Sciences (SAMS) [47]. Following the SAMS guideline by analogy, the three principles of research ethics—respect for individuals, welfare and justice—will be given special attention in this study.

## Discussion

This protocol is designed to develop an interprofessional educational intervention and to examine it in everyday care. If the results of this feasibility study are positive a larger study will be conducted. This larger study will generate new information on the knowledge, attitudes and behaviors of patients with VLU in regards to adherence to treatment recommendations and to what effect these can be modified by an interdisciplinary educational intervention. The results will subsequently inform the development/review of current clinical guidelines. Enhancing patient adherence to therapy is a challenge that is not unique to the care of patients with VLU or the field of wound care. The findings can be applied to patients with other chronic conditions that require close adherence to treatment regimes Additional file 1.

## Additional file

Additional file 1: Standard protocol items: recommendation for interventional trials (SPIRIT) 2013 checklist: recommended items to address in a clinical trial protocol and related documents*. (DOC 121 kb)

## References

[1] Persoon A, Heinen MM, van der Vleuten CJ, de Rooij MJ, van de Kerkhof PC, van Achterberg T (2004). Leg ulcers: a review of their impact on daily life. J Clin Nurs.

[2] Heinen MM, van Achterberg T, op Reimer WS, van de Kerkhof PC, de Laat E (2004). Venous leg ulcer patients: a review of the literature on lifestyle and pain-related interventions. J Clin Nurs.

[3] Graham ID, Harrison MB, Nelson EA, Lorimer K, Fisher A (2003). Prevalence of lower-limb ulceration: a systematic review of prevalence studies. Adv Skin Wound Care.

[4] Harrisson MB, Graham ID, Friedberg E, Lorimer K, Vandevelde CS (2001). Assessing the population with leg and foot ulcers. Canadian Nurse.

[5] Abbade LP, Lastoria S, de Almeida Rollo H, Stolf HO (2005). A sociodemographic, clinical study of patients with venous ulcer. Int J Dermatol.

[6] McDaniel HB, Marston WA, Farber MA, Mendes RR, Owens LV, Young ML (2002). Recurrence of chronic venous ulcers on the basis of clinical, etiologic, anatomic, and pathophysiologic criteria and air plethysmography. J Vasc Surg.

[7] Finlayson Kathleen, Wu Min-Lin, Edwards Helen E. (2015). Identifying risk factors and protective factors for venous leg ulcer recurrence using a theoretical approach: A longitudinal study. International Journal of Nursing Studies.

[8] Saha SP, Whayne TF, Mukherjee DP (2011). Current management of peripheral vascular disease: where is the evidence?. Cardiovasc Hematol Agents Med Chem.

[9] Posnett J, Gottrup F, Lundgren H, Saal G (2009). The resource impact of wounds on health-care providers in Europe. J Wound Care.

[10] Swiss Federal Statistical Office. Population - key-figures 2015. Available from: https://www.bfs.admin.ch/bfs/en/home/statistics/population/effectif-change.html. Accessed 17 Nov 2017.

[11] Swiss Federal Statistical Office. Costs and financing of the health care system 2017. Available from: https://www.bfs.admin.ch/bfs/en/home/statistics/health/costs-financing.html. Accessed 17 Nov 2017.

[12] Australian and New Zealand Wound Managment Associations (2011). Australian and New Zealand Clinical practive guideline for prevention and managment of venous leg ulcers.

[13] Association for the Advancement of Wound Care (2010). Association for the Advancement of Wound Care (AAWC) venous ulcer guideline.

[14] SIGN (GB) - Scottish Intercollegiate Guidelines Network (2010). Managment of chronic venous leg ulcers (SIGN CPG 120).

[15] National Institute for Health and Care Excellence (NICE) (2013). Varicose veins in the legs. The diagnosis and management of varicose veins.

[16] Franks P, Barker J, Collier M, Gethin G, Haesler E, Jawien A (2016). Management of patients with venous leg ulcer: challenges and current best practice. J Wound Care.

[17] Van Hecke A, Grypdonck M, Defloor T (2008). Interventions to enhance patient compliance with leg ulcer treatment: a review of the literature. J Clin Nurs.

[18] Moffatt C, Kommala D, Dourdin N, Choe Y (2009). Venous leg ulcers: patient concordance with compression therapy and its impact on healing and prevention of recurrence. Int Wound J.

[19] Edwards LM, Moffatt CJ, Franks PJ (2002). An exploration of patients' understanding of leg ulceration. J Wound Care.

[20] Finlayson K, Edwards H, Courtney M (2010). The impact of psychosocial factors on adherence to compression therapy to prevent recurrence of venous leg ulcers. J Clin Nurs.

[21] Van Hecke A, Grypdonck M, Beele H, Vanderwee K, Defloor T (2011). Adherence to leg ulcer lifestyle advice: qualitative and quantitative outcomes associated with a nurse-led intervention. J Clin Nurs.

[22] Heinen MM, Persoon A, van de Kerkhof P, Otero M, van Achterberg T (2007). Ulcer-related problems and health care needs in patients with venous leg ulceration: a descriptive, cross-sectional study. Int J Nurs Stud.

[23] Heinen M, Borm G, van der Vleuten C, Evers A, Oostendorp R, van Achterberg T (2012). The Lively Legs self-management programme increased physical activity and reduced wound days in leg ulcer patients: results from a randomized controlled trial. Int J Nurs Stud.

[24] Brooks J, Ersser SJ, Lloyd A, Ryan TJ (2004). Nurse-led education sets out to improve patient concordance and prevent recurrence of leg ulcers. J Wound Care.

[25] Patient information: chronic venous disease (beyond the basics) [Internet]. UptoDate. 2016 [cited 07.16.2016]. Available from: https://www.uptodate.com/contents/chronic-venous-disease-beyond-the-basics.

[26] Borg GA (1982). Psychophysical bases of perceived exertion. Med Sci Sports Exerc.

[27] Van Hecke A, Verhaeghe S, Grypdonck M, Beele H, Flour M, Defloor T (2011). Systematic development and validation of a nursing intervention: the case of lifestyle adherence promotion in patients with leg ulcers. J Adv Nurs.

[28] Kelechi TJ, Mueller M, Spencer C, Rinard B, Loftis G (2014). The effect of a nurse-directed intervention to reduce pain and improve behavioral and physical outcomes in patients with critically colonized/infected chronic leg ulcers. J Wound Ostomy Continence Nurs.

[29] Casey G (2003). Nutritional support in wound healing. Nurs Stand.

[30] MacKay D, Miller AL (2003). Nutritional support for wound healing. Altern Med Rev.

[31] McDaniel Jodi C., Kemmner Kaitlyn G., Rusnak Sarah (2015). Nutritional profile of older adults with chronic venous leg ulcers: A pilot study. Geriatric Nursing.

[32] Milne AC, Potter J, Vivanti A, Avenell A (2009). Protein and energy supplementation in elderly people at risk from malnutrition. Cochrane Database Syst Rev.

[33] Wilkinson EA (2014). Oral zinc for arterial and venous leg ulcers. Cochrane Database Syst Rev.

[34] Van Hecke A, Verhaeghe S, Grypdonck M, Beele H, Defloor T (2011). Processes underlying adherence to leg ulcer treatment: a qualitative field study. Int J Nurs Stud.

[35] Keller-Senn A, Probst S, Mahrer Imhof R, Imhof L (2015). Nurse-led education programme enhancing foot care self-efficacy in high-risk diabetes population. Int Diabetes Nurs.

[36] Lee JY, Yoo KH, Kim DK, Kim SH, Kim TE, Kim TH (2016). Effects of educational interventions for chronic airway disease on primary care. J Korean Med Sci.

[37] Bobridge A, Sandison S, Paterson J, Puckridge P, Esplin M (2011). A pilot study of the development and implementation of a 'best practice' patient information booklet for patients with chronic venous insufficiency. Phlebology..

[38] O'Brien JA, Finlayson KJ, Kerr G, Edwards HE (2014). Testing the effectiveness of a self-efficacy based exercise intervention for adults with venous leg ulcers: protocol of a randomised controlled trial. BMC Dermatol.

[39] Klonizakis M, Tew GA, Gumber A, Crank H, King B, Middleton G (2018). Supervised exercise training as an adjunct therapy for venous leg ulcers: a randomized controlled feasibility trial. Br J Dermatol.

[40] European Wound Management Association. Position document-wound bed preperation in practice. London: 2004. https://ewma.org/resources/for-professionals/ewma-documents-and-joint-publications/ewma-position-documents-2002-2008/. Accessed 14 Apr 2019.

[41] Kan YM, Delis KT (2001). Hemodynamic effects of supervised calf muscle exercise in patients with venous leg ulceration: a prospective controlled study. Arch Surg.

[42] Yang D, Vandongen YK, Stacey MC (1999). Effect of exercise on calf muscle pump function in patients with chronic venous disease. Br J Surg.

[43] O'Brien J, Edwards H, Finlayson K, Kerr G (2002). Understanding the relationships between the calf muscle pump, ankle range of motion and healing for adults with venous leg ulcers: a review of the literature. Wound Pract Res.

[44] Chodzko-Zajko WJ, Proctor DN, Fiatarone Singh MA, Minson CT, Nigg CR, Salem GJ (2009). American College of Sports Medicine position stand. Exercise and physical activity for older adults. Med Sci Sports Exerc.

[45] Kaewkannate K, Kim S (2016). A comparison of wearable fitness devices. BMC Public Health.

[46] Widmer LK, Stähelin HB, Nissen C, Da Silva A (1981). Venen-, arterien-krankheiten, koronare herzkrankheiten bei berufstätigen.

[47] Schweizerische Akademie der Medizinischen Wissenschaften. Forschung mit menschen - ein leitfaden für die praxis. Basel: 2009. https://www.samw.ch/de/Projekte/Archiv/Forschung-mit-Menschen.html. Accessed 14 Apr 2019.

